# Supplementary material for: A two-kinesin mechanism controls neurogenesis in the developing brain
Source: Commun Biol. 2023 Dec 1;6:1219. doi: 10.1038/s42003-023-05604-5 (PMC10692124; doi:10.1038/s42003-023-05604-5)
Supplement: Supplementary file 2 — Supplemental Figures [file 42003_2023_5604_MOESM2_ESM.pdf]

Figure S1. Kif1a shRNA increases Pax6+/TBR2+ cell number

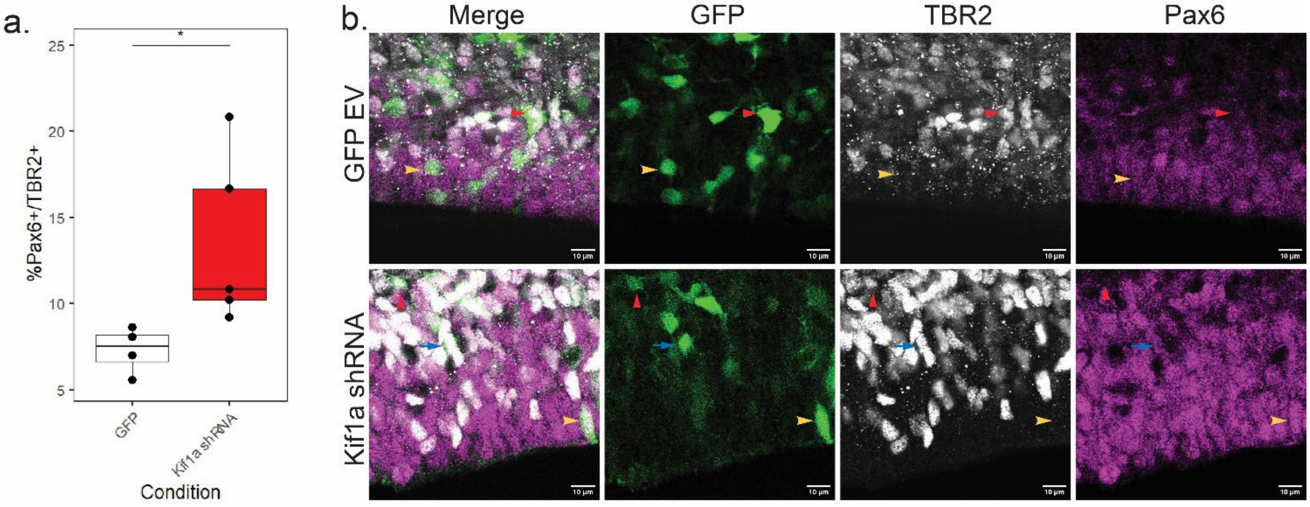


(a) Quantification of Pax6+/TBR2+ cells in E19 rat brains. Data plotted as interquartile range with 5-95% whisker range. (*$P < 0.05$. Analyzed using two sided unpaired t-test. Total brains for GFP control = 4, Kif1a shRNA = 5. At least 46 cells per brain were included).

(b) Representative images of Pax6/TBR2 double staining in E19 rat brains in GFP EV (top) and Kif1a shRNA (bottom). Red arrows indicate GFP+/Pax6-/TBR2+ cells. Yellow arrows indicate GFP+/Pax6+/TBR2- cells. Blue arrows in Kif1a shRNA condition indicate an example of a GFP+/Pax6+/TBR2+ cell. Scale bar = 10 µm

Figure S2. Conditional knockout of Kif13B in mouse shows similar phenotype to Kif13b shRNA expression in rat brain.

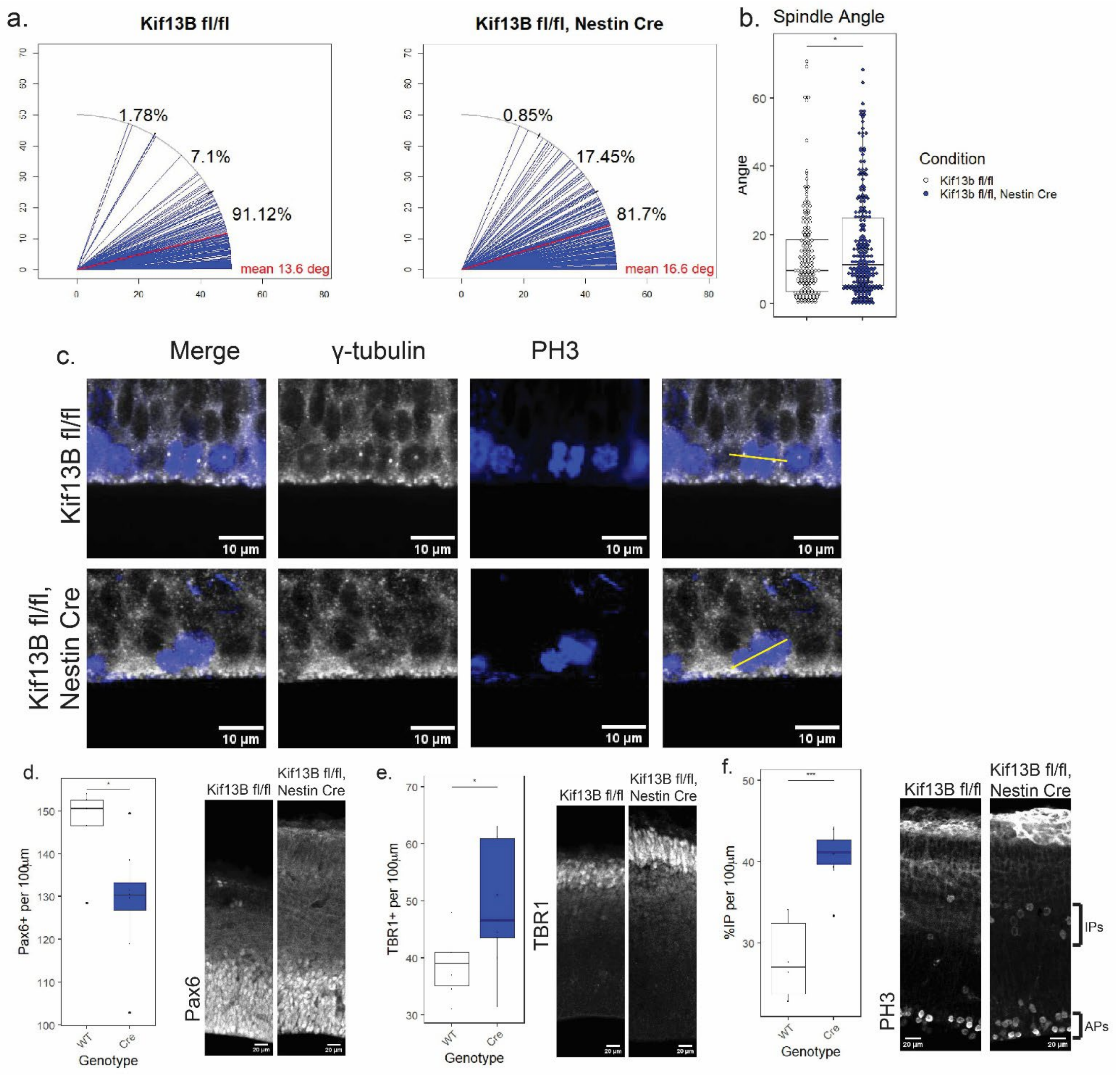

(a) Plots of spindle angle in E14.5 mouse brain with Kif13B fl/fl and either no Cre control (left) or Nestin Cre (right). Mean spindle angle relative to the ventricular surface for each condition is shown in red. Percentages shown in black show percent of cells with spindle angles in 0-30, 30-60, and 60-90 degree range. (Total cells from Kif13B fl/fl control = 169 cells from 7 brains, Kif13B fl/fl, Nestin Cre = 235 cells from 9 brains).

(b) Plot of spindle angles in Kif13B fl/fl (white) and Kif13B fl/fl, Nestin Cre (blue) conditions. Data plotted as interquartile range with 5-95% whisker range. (*P < 0.05, analyzed using one sided unpaired t-test.)

(c) Representative image of dividing RGPs in Kif13B fl/fl and Kif13B fl/fl, Nestin Cre mice at E14.5. Gamma tubulin is shown in gray, PH3 in blue. A yellow line indicates the spindle angle relative to the ventricular surface. Scale bar = 10 µm.

(d-e) Quantification of cell markers in E14.5 mice. Markers were quantified in a 100µm wide section of the cortex for Kif13B fl/fl (WT, white) or Kif13B fl/fl, Nestin Cre (Cre, blue), and total number of cells positive for Pax6 (d) or TBR1 (e) were calculated. Data plotted as interquartile range with 5-95% whisker range. PH3 (f) was calculated as the percent of PH3+ cells dividing at a distance from the ventricular surface. Representative images for each staining are shown. Brackets to the right of images in (f) indicate distinct regions of PH3+ cells, with IPs appearing farther from the ventricular surface and APs appearing adjacent to the ventricle. Scale bar = 20 µm (*P < 0.05. ***P < 0.001, analyzed using two sided unpaired t-test. Total brains for Kif13B fl/fl control = 6, Kif13B fl/fl, Nestin Cre = 9. At least 40 cells for each brain were included for analysis in (f))

Figure S3. Double expression of Kif1a shRNA and Kif13b shRNA partially rescues both phenotypes

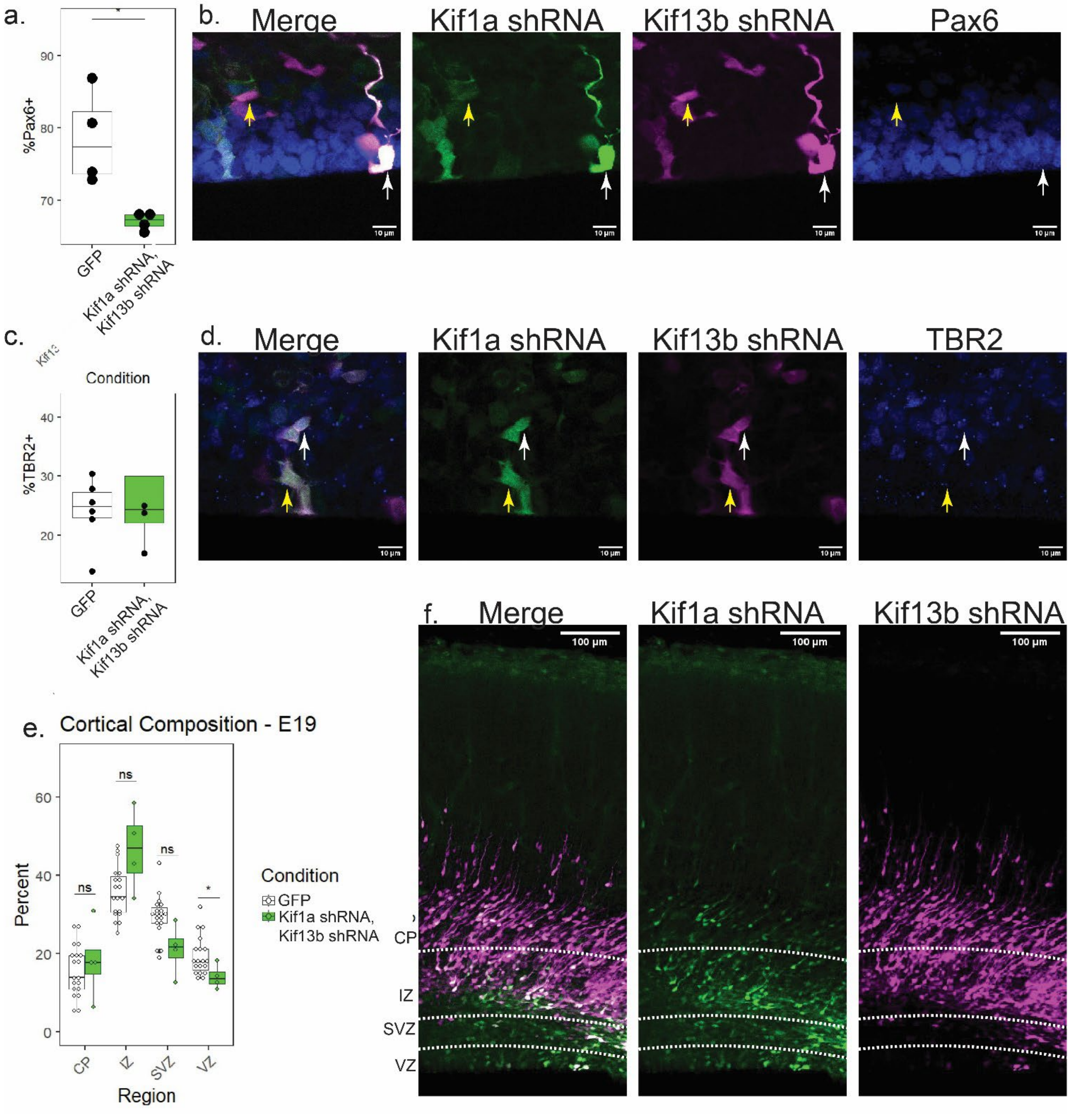


(a) Quantification of Pax6+ cells in E19 rat brain VZ. The percentage of Pax6+ cells was calculated as number of GFP+/Pax6+ or GFP+/RFP+/Pax6+ cells divided by total GFP+ or

GFP+/RFP+ cells at the ventricular surface. Data plotted as interquartile range with 5-95% whisker range. (*P < 0.05, Analyzed using two sided unpaired t-test. Total brains for GFP control = 5, Kif1a shRNA + Kif1a shRNA = 4. At least 81 cells from each brain were included).

(b) Representative images of Kif1a shRNA (green) + Kif13b shRNA expression (magenta) and Pax6 staining (blue). White arrows indicate GFP+/RFP+/Pax6+ cells and yellow arrows indicate GFP+/RFP+/Pax6- cells. Scale bar = 10 µm

(c) Quantification of TBR2+ cells in E19 rat brain VZ. The percentage of TBR2+ cells was calculated as number of GFP+/ TBR2 + or GFP+/RFP+/ TBR2 + cells divided by total GFP+ or GFP+/RFP+ cells at the ventricular surface. Data plotted as interquartile range with 5-95% whisker range. (Analyzed using two sided unpaired t-test. Total brains for GFP control = 5, Kif1a shRNA + Kif1a shRNA = 4. At least 52 cells per brain were included).

(d) Representative images of Kif1a shRNA (green) + Kif13b shRNA expression (magenta) and TBR2 staining (blue). White arrows indicate GFP+/RFP+/ TBR2 + cells and yellow arrows indicate GFP+/RFP+/ TBR2 - cells. Scale bar = 10 µm

(e) Percent of cells in CP, IZ, SVZ, or VZ of the cortex at E19. Data plotted as interquartile range with 5-95% whisker range. (*P < 0.05, Analyzed using two sided unpaired t-test. Total GFP control brains: 19. Total Kif1a shRNA + Kif13b shRNA brains: 4. At least 118 cells from each brain were included).

(f) Representative image of an E19 rat brain expressing Kif1a shRNA (green) and Kif13b shRNA (magenta). White dashed lines indicate the borders between cortical regions. Scale bar = 100 µm.

Figure S4. Overexpression of Kif13B or Kif1A results in little change to cortical development

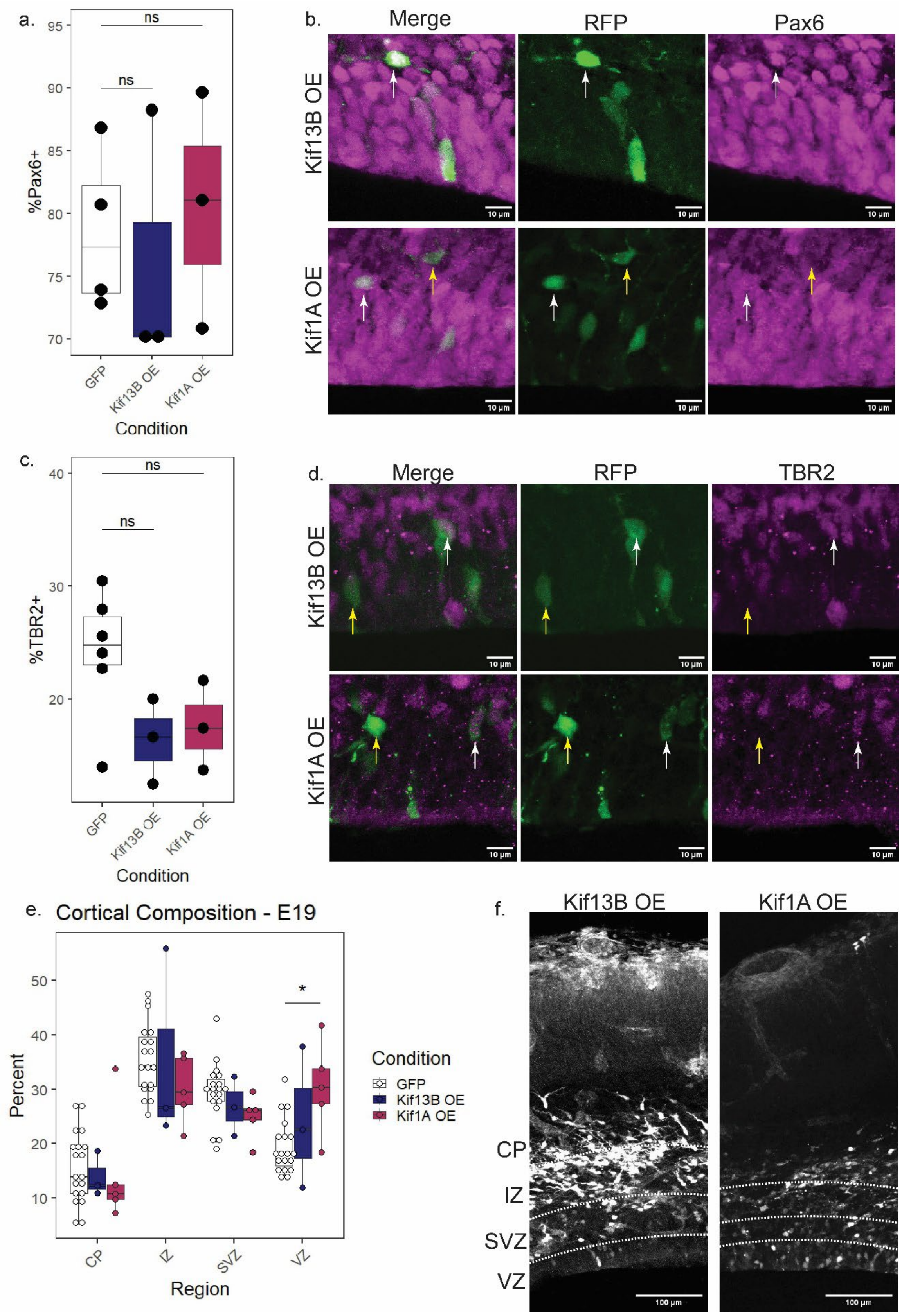

(a) Quantification of Pax6+ cells in E19 rat brain VZ. The percentage of Pax6+ cells was calculated as number of RFP+/Pax6+ cells divided by total RFP+ cells at the ventricular surface. Data plotted as interquartile range with 5-95% whisker range. (*$P < 0.05$, Analyzed using two sided unpaired t-test. Total brains for GFP control = 5, Kif13B OE = 3, Kif1A OE = 3. At least 17 cells from each brain were included).

(a) Representative images of Kif13B or Kif1A OE (green) and Pax6 staining (magenta). White arrows indicate RFP+/Pax6+ cells and yellow arrows indicate RFP+/Pax6- cells. Scale bar = 10 μm

(c) Quantification of TBR2+ cells in E19 rat brain VZ. The percentage of TBR2+ cells was calculated as number of RFP+/ TBR2 + cells divided by total GFP+ or GFP+/RFP+ cells at the ventricular surface. Data plotted as interquartile range with 5-95% whisker range. (Analyzed using two sided unpaired t-test. Total brains for GFP control = 5, Kif13B OE = 3, Kif1A OE = 3. At least 20 cells from each brain were included).

(d) Representative images of Kif13B or Kif1A OE (green) and TBR2 staining (magenta). White arrows indicate RFP+/ TBR2 + cells and yellow arrows indicate RFP+/ TBR2- cells. Scale bar = 10 μm.

(e) Percent of cells in CP, IZ, SVZ, or VZ of the cortex at E19. Data plotted as interquartile range with 5-95% whisker range. (*$P < 0.05$, Analyzed using two sided unpaired t-test. Total GFP control brains = 19. Total Kif13B OE brains = 3. Total Kif1A OE brains = 4. At least 90 cells from each brain were included).

(f) Representative images of E19 rat brains expressing Kif13B OE or Kif1A OE. White dashed lines indicate the border between cortical regions. Scale bar = 100 μm.

Figure S5. Overexpression of Kif1A or Kif13B, or knockdown of both Kif1A and Kif13B affect RGP mitosis

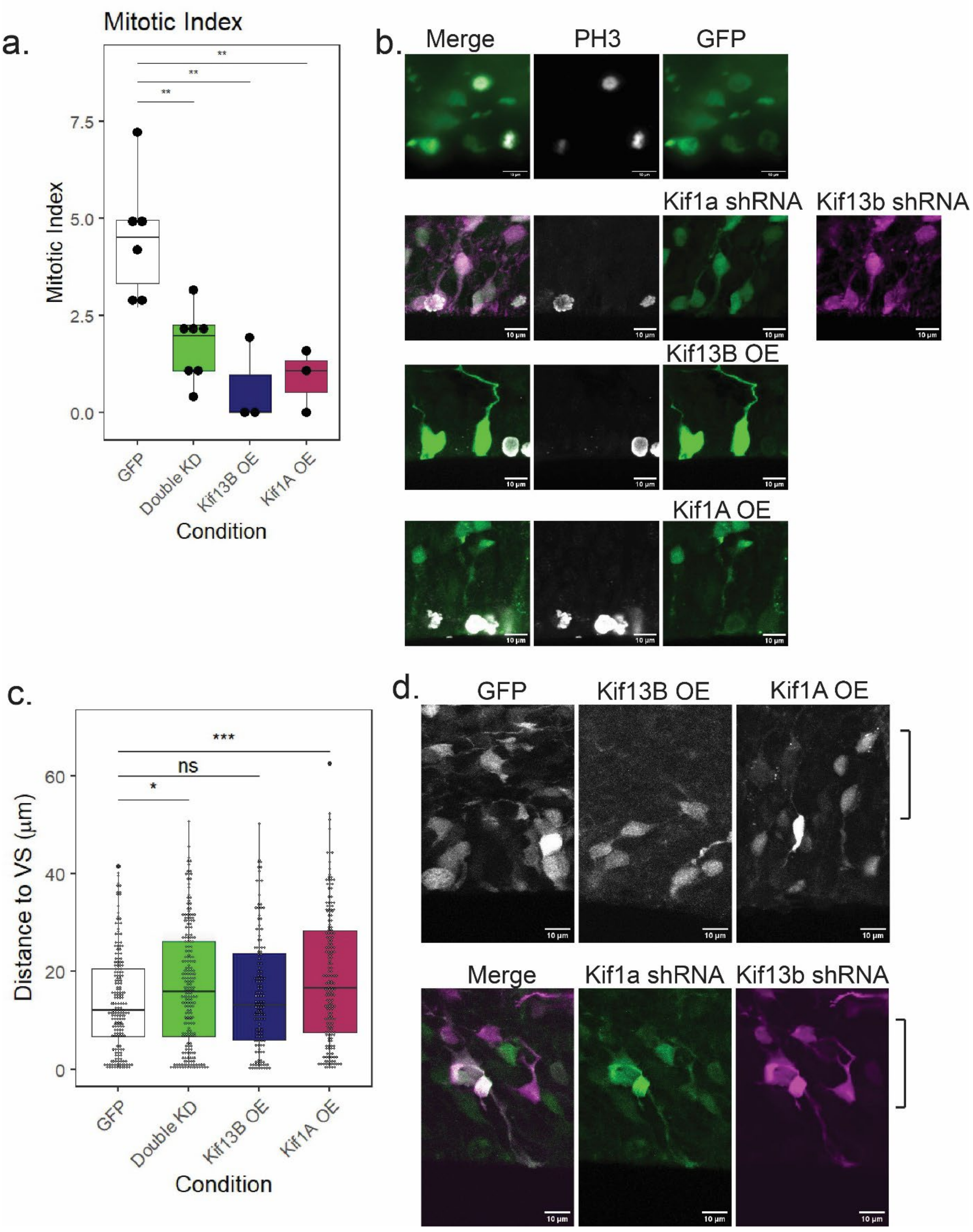

(a) Quantification of mitotic index in RGPs expressing GFP control, Kif1a shRNA + Kif13b shRNA (Double KD), Kif13B OE, or Kif1A OE. Mitotic index was quantified as the percent of GFP+/PH3+ (control), GFP+/RFP+/PH3+ (Double KD), or RFP+/PH3+ (Kif13B OE and Kif1A OE) cells compared to the total number of transfected cells in the VZ in each condition. Data plotted as interquartile range with 5-95% whisker range. (**$P < 0.01$, Analyzed using two sided unpaired t-test. Total brains for GFP control = 6, Double KD = 7, Kif13B OE = 3, Kif1A OE = 3. At least 31 cells from each brain were included).

(b) Representative images of GFP control, Kif1a shRNA, Kif13B OE, or Kif1A OE (green) and Kif13b shRNA (magenta) with PH3 (white). Scale bar = 10 µm.

(c) Quantification of distance from apical end of cell body to the ventricular surface in RGPs expressing GFP control, Kif1a shRNA + Kif13b shRNA (Double KD), Kif13B OE, or Kif1A OE. Data plotted as interquartile range with 5-95% whisker range. (*$P < 0.05$, ***$P < 0.001$. Analyzed using two sided unpaired t-test. Total brains for GFP control = 5, Double KD = 4, Kif13B OE = 3, Kif1A OE = 3. At least 27 cells from each brain were included).

(d) Representative images of GFP control, Kif13B OE, Kif1A OE, and Double KD RGPs. Brackets to the right of Kif1A OE and Double KD images show the region roughly 30-50 µm from the ventricular surface where cells are not often seen in GFP control or Kif13B OE, and a large proportion of cells are seen in the Kif1A OE and Double KD conditions. Scale bar = 10 µm.
