# Supplementary material for: A two-kinesin mechanism controls neurogenesis in the developing brain
Source: Commun Biol. 2023 Dec 1;6:1219. doi: 10.1038/s42003-023-05604-5 (PMC10692124; doi:10.1038/s42003-023-05604-5)
Supplement: Supplementary file 3 — Description of Additional Supplementary Files [file 42003_2023_5604_MOESM3_ESM.docx]

Description of Additional Supplementary Files

**File name:** Supplementary Data 1

**Description:** All data included for analysis in this study are included in Supplementary Data 1. It is presented as an Excel file, with an individual sheet for each figure in the study. All the data for each figure are presented in the relevant sheet.
