# Supplementary material for: A two-kinesin mechanism controls neurogenesis in the developing brain
Source: Commun Biol. 2023 Dec 1;6:1219. doi: 10.1038/s42003-023-05604-5 (PMC10692124; doi:10.1038/s42003-023-05604-5)
Supplement: Supplementary file 5 — Reporting Summary [file 42003_2023_5604_MOESM5_ESM.pdf]

nature portfolio

Corresponding author(s): Richard Vallee, Paige Helmer

Last updated by author(s): Oct 19, 2023

Reporting Summary

Nature Portfolio wishes to improve the reproducibility of the work that we publish. This form provides structure for consistency and transparency in reporting. For further information on Nature Portfolio policies, see our Editorial Policies and the Editorial Policy Checklist.

Statistics

For all statistical analyses, confirm that the following items are present in the figure legend, table legend, main text, or Methods section.

n/a	Confirmed	
☐	☒	The exact sample size (*n*) for each experimental group/condition, given as a discrete number and unit of measurement
☐	☒	A statement on whether measurements were taken from distinct samples or whether the same sample was measured repeatedly
☐	☒	The statistical test(s) used AND whether they are one- or two-sided<br>*Only common tests should be described solely by name; describe more complex techniques in the Methods section.*
☐	☒	A description of all covariates tested
☐	☒	A description of any assumptions or corrections, such as tests of normality and adjustment for multiple comparisons
☐	☒	A full description of the statistical parameters including central tendency (e.g. means) or other basic estimates (e.g. regression coefficient) AND variation (e.g. standard deviation) or associated estimates of uncertainty (e.g. confidence intervals)
☐	☒	For null hypothesis testing, the test statistic (e.g. *F*, *t*, *r*) with confidence intervals, effect sizes, degrees of freedom and *P* value noted<br>*Give P values as exact values whenever suitable.*
☒	☐	For Bayesian analysis, information on the choice of priors and Markov chain Monte Carlo settings
☒	☐	For hierarchical and complex designs, identification of the appropriate level for tests and full reporting of outcomes
☒	☐	Estimates of effect sizes (e.g. Cohen's *d*, Pearson's *r*), indicating how they were calculated

Our web collection on statistics for biologists contains articles on many of the points above.

Software and code

Policy information about availability of computer code

Data collection	FV10-ASW Viewer Software version 04.01 was used to acquire images.
Data analysis	ImageJ version 2.14.0 was used for image analysis. Fluorender version 2.28 was used for spindle orientation analysis. R code provided in Jüschke, C., Xie, Y., Postiglione, M. P. & Knoblich, J. A. Analysis and modeling of mitotic spindle orientations in three dimensions. PNAS 111, 1014–1019 (2014) was used for analysis of spindle orientation. R version 4.3.1 with the ggplot2 package was used for statistical analysis and to generate graphs. GraphPad Prism version 10.0.3 was used for statistical analysis.

For manuscripts utilizing custom algorithms or software that are central to the research but not yet described in published literature, software must be made available to editors and reviewers. We strongly encourage code deposition in a community repository (e.g. GitHub). See the Nature Portfolio guidelines for submitting code & software for further information.

Data

Policy information about availability of data

All manuscripts must include a data availability statement. This statement should provide the following information, where applicable:

- Accession codes, unique identifiers, or web links for publicly available datasets
- A description of any restrictions on data availability
- For clinical datasets or third party data, please ensure that the statement adheres to our policy

No datasets requiring submission to a data repository were generated in this study. All other data are included in the paper.

Research involving human participants, their data, or biological material

Policy information about studies with human participants or human data. See also policy information about sex, gender (identity/presentation), and sexual orientation and race, ethnicity and racism.

Reporting on sex and gender	*Use the terms sex (biological attribute) and gender (shaped by social and cultural circumstances) carefully in order to avoid confusing both terms. Indicate if findings apply to only one sex or gender; describe whether sex and gender were considered in study design; whether sex and/or gender was determined based on self-reporting or assigned and methods used. Provide in the source data disaggregated sex and gender data, where this information has been collected, and if consent has been obtained for sharing of individual-level data; provide overall numbers in this Reporting Summary. Please state if this information has not been collected. Report sex- and gender-based analyses where performed, justify reasons for lack of sex- and gender-based analysis.*
Reporting on race, ethnicity, or other socially relevant groupings	*Please specify the socially constructed or socially relevant categorization variable(s) used in your manuscript and explain why they were used. Please note that such variables should not be used as proxies for other socially constructed/relevant variables (for example, race or ethnicity should not be used as a proxy for socioeconomic status). Provide clear definitions of the relevant terms used, how they were provided (by the participants/respondents, the researchers, or third parties), and the method(s) used to classify people into the different categories (e.g. self-report, census or administrative data, social media data, etc.) Please provide details about how you controlled for confounding variables in your analyses.*
Population characteristics	*Describe the covariate-relevant population characteristics of the human research participants (e.g. age, genotypic information, past and current diagnosis and treatment categories). If you filled out the behavioural & social sciences study design questions and have nothing to add here, write "See above."*
Recruitment	*Describe how participants were recruited. Outline any potential self-selection bias or other biases that may be present and how these are likely to impact results.*
Ethics oversight	*Identify the organization(s) that approved the study protocol.*

Note that full information on the approval of the study protocol must also be provided in the manuscript.

Field-specific reporting

Please select the one below that is the best fit for your research. If you are not sure, read the appropriate sections before making your selection.

☒ Life sciences ☐ Behavioural & social sciences ☐ Ecological, evolutionary & environmental sciences

For a reference copy of the document with all sections, see nature.com/documents/nr-reporting-summary-flat.pdf

Life sciences study design

All studies must disclose on these points even when the disclosure is negative.

Sample size	No statistical method was employed to predetermine sample size. As is common practice, at least 3 embryos from at least 2 different pregnant dams were analyzed per age/condition. Statistical significance was determined using t-tests.
Data exclusions	No data were excluded
Replication	All experiments were replicated at least 3 times
Randomization	Mouse breedings were set up with one parent with no Nestin Cre allele, and the other parent heterozygous for Nestin Cre. This resulted in roughly 50% of mouse embryos in each litter inheriting Nestin Cre and 50% without Nestin Cre, providing randomization within each litter. Rat experiments were all performed on a WT background and not randomized.
Blinding	Analysis of embryonic mouse brains was performed blinded with the genotype of each individual determined after analysis was complete. Blinding was not possible for IUE experiments, as the position of each embryo with the constructs injected had to be recorded at the time of injection, and author PH performed all experiments alone.

Reporting for specific materials, systems and methods

We require information from authors about some types of materials, experimental systems and methods used in many studies. Here, indicate whether each material, system or method listed is relevant to your study. If you are not sure if a list item applies to your research, read the appropriate section before selecting a response.

Materials & experimental systems

n/a	Involved in the study
☐	☒ Antibodies
☒	☐ Eukaryotic cell lines
☒	☐ Palaeontology and archaeology
☐	☒ Animals and other organisms
☒	☐ Clinical data
☒	☐ Dual use research of concern
☒	☐ Plants

Methods

n/a	Involved in the study
☒	☐ ChIP-seq
☒	☐ Flow cytometry
☒	☐ MRI-based neuroimaging

Antibodies

Antibodies used	PH3 (Abcam, ab14955, mouse, 1:750); Pax6 (Covance, PRB-278p, rabbit, 1:300); gamma tubulin (Proteintech, 15176-1-AP, rabbit, 1:300); TBR2 (Abcam, ab23345, rabbit, 1:750); TBR1 (Abcam, ab31940, rabbit, 1:200); Pax6 (Abcam, ab78545, mouse, 1:100)
Validation	All antibodies were commercially produced and validated by the company they were purchased from.

Animals and other research organisms

Policy information about studies involving animals; ARRIVE guidelines recommended for reporting animal research, and Sex and Gender in Research

Laboratory animals	Timed pregnant Sprague-Dawley WT rats were purchased from Taconic or Charles River. Kif13B flox mouse sperm was provided by the Bolino lab and cryorecovered at Charles River. Nestin Cre mice were purchased from Jackson Labs. All mouse models were on a C57BL/6 genetic background.
Wild animals	No wild animals were used
Reporting on sex	Embryonic animals were used, at a stage where sex is not visibly determinable and thus information on the sex of the animals was not collected.
Field-collected samples	No field-collected samples were used
Ethics oversight	The Columbia University IACUC reviewed and approved all animal protocols

Note that full information on the approval of the study protocol must also be provided in the manuscript.

Plants

Seed stocks	*Report on the source of all seed stocks or other plant material used. If applicable, state the seed stock centre and catalogue number. If plant specimens were collected from the field, describe the collection location, date and sampling procedures.*
Novel plant genotypes	*Describe the methods by which all novel plant genotypes were produced. This includes those generated by transgenic approaches, gene editing, chemical/radiation-based mutagenesis and hybridization. For transgenic lines, describe the transformation method, the number of independent lines analyzed and the generation upon which experiments were performed. For gene-edited lines, describe the editor used, the endogenous sequence targeted for editing, the targeting guide RNA sequence (if applicable) and how the editor was applied.*
Authentication	*Describe any authentication procedures for each seed stock used or novel genotype generated. Describe any experiments used to assess the effect of a mutation and, where applicable, how potential secondary effects (e.g. second site T-DNA insertions, mosiacism, off-target gene editing) were examined.*
